# Heart rate variability: a tool to explore the sleeping brain?

**DOI:** 10.3389/fnins.2014.00402

**Published:** 2014-12-11

**Authors:** Florian Chouchou, Martin Desseilles

**Affiliations:** ^1^NeuroPain Unit, Lyon Neuroscience Research Centre, CRNL – INSERM U 1028/CNRS UMR 5292, University of LyonFrance; ^2^Department of Psychology, University of NamurNamur, Belgium; ^3^Cyclotron Research Centre, University of LiègeLiège, Belgium

**Keywords:** Sleep, heart rate variability, ANS, REM sleep, Non-REM sleep, emotion

## Abstract

Sleep is divided into two main sleep stages: (1) non-rapid eye movement sleep (non-REMS), characterized among others by reduced global brain activity; and (2) rapid eye movement sleep (REMS), characterized by global brain activity similar to that of wakefulness. Results of heart rate variability (HRV) analysis, which is widely used to explore autonomic modulation, have revealed higher parasympathetic tone during normal non-REMS and a shift toward sympathetic predominance during normal REMS. Moreover, HRV analysis combined with brain imaging has identified close connectivity between autonomic cardiac modulation and activity in brain areas such as the amygdala and insular cortex during REMS, but no connectivity between brain and cardiac activity during non-REMS. There is also some evidence for an association between HRV and dream intensity and emotionality. Following some technical considerations, this review addresses how brain activity during sleep contributes to changes in autonomic cardiac activity, organized into three parts: (1) the knowledge on autonomic cardiac control, (2) differences in brain and autonomic activity between non-REMS and REMS, and (3) the potential of HRV analysis to explore the sleeping brain, and the implications for psychiatric disorders.

## Introduction

The autonomic nervous system (ANS) connects the body's nervous system to the main physiological systems, and is largely modulated by reflex loops, the hypothalamic and brainstem centers, and the afferent and efferent pathways. For example, the baroreflex and chemoreflex loops—both autonomic cardiovascular reflexes—involve pathways from baroreceptors and chemoreceptors to central processes and subsequently the sympathetic and parasympathetic motor arms (Guyenet, 2013). However, the concept has been extended to include higher central nervous system centers, whereby modulation of higher brain structures mediates cardiovascular responses. Brain imaging and electrophysiological studies have demonstrated the involvement of certain subcortical and cortical regions (for a review, see Beissner et al., 2013), including the amygdala and the midcingulate and insular cortices, enabling integration of simple (e.g., sensory) and complex (e.g., emotional) information in autonomic cardiovascular activity (Critchley and Harrison, 2013).

Heart rate variability (HRV) analysis, used to assess autonomic cardiac activity, highlighted higher parasympathetic tone during non-rapid eye movement sleep (non-REMS) compared to a sympathovagal balance shift from parasympathetic predominance toward sympathetic hyperactivity during rapid eye movement sleep (REMS) (Mendez et al., 2006; Cabiddu et al., 2012). Moreover, REMS and non-REMS were linked to differential brain activity: non-REMS is characterized by slow EEG rhythms such as delta wave, with events such as sleep spindles and K-complexes, associated with lower brain activity compared to wakefulness; whereas REMS is characterized by low-amplitude, high-frequency EEG rhythms, rapid eye movements (REM), and muscular atonia despite global brain activity similar to wakefulness (called “paradoxical” sleep) (Desseilles et al., 2008, 2011b; Dang-Vu et al., 2010; Dang-Vu, 2012).

To address whether brain activity modulation during sleep contributes to changes in autonomic cardiac modulation from non-REMS to REMS, we develop three points: (1) the current knowledge on autonomic cardiac control, (2) differences in cerebral and autonomic activity between non-REMS and REMS, and (3) using HRV analysis to explore the sleeping brain, and implications for psychiatric disorders.

## Technical considerations

Cardiac activity is controlled by the sympathetic and parasympathetic systems (Guyenet, 2013), which induce heart rate oscillations at different rhythms. Mathematical methods (e.g., time- and frequency-domain analysis) are used to study these rhythms and consequently autonomic cardiac modulations, including time- and frequency-domain analysis (Rajendra Acharya et al., 2006). In this mini-review, we focus on the most frequent methods for exploring autonomic cardiac modulation in combination with brain imaging [functional magnetic resonance imaging (fMRI) or positron emission tomography scan (PET scan)]. We excluded long-term heart rate (HR) oscillations due to debatable physiological interpretations and irrelevance to the study question (more than 5 min).

### Time-domain analysis

This method describes HR using a mean or standard deviation. The standard deviation of normal-to-normal intervals (SDNN) represents the variability over the entire recording period, obtaining the *overall* autonomic modulation regardless of sympathetic or parasympathetic arm (Rajendra Acharya et al., 2006). Other indices describe parasympathetic tone, calculated from differences between consecutive heart beats, representing short-term variability (European Society of Cardiology, North American Society of Pacing and Electrophysiology, 1996). These measures include the root mean square successive difference (rMSSD), number of interval differences of successive heart beats greater than 50 ms (NN50), and proportion of NN50 (pNN50, NN50 divided by total number of heart beats).

### Frequency-domain analysis: fourier transforms

The Fourier transform decomposes a function according to its contained frequencies to build a spectral power spectrum for each frequency. To examine autonomic cardiac modulation in an HR Fourier spectrum, total spectral power (0–0.4 Hz) is considered (low-frequency—LF, 0.04–0.15 Hz; high-frequency—HF, 0.15–0.4 Hz) (European Society of Cardiology, North American Society of Pacing and Electrophysiology, 1996; Rajendra Acharya et al., 2006).

Total spectral power indicates overall HRV and allows assessing overall autonomic cardiac modulation (e.g., SDNN). HF power represents short-term HR variation. Studies showed that injected atropine completely eliminated HF power (Akselrod et al., 1981; Pomeranz et al., 1985). Thus, HF power is modulated by parasympathetic activity only, corresponding to peak respiratory rate (0.18–0.40 Hz). Pharmacological studies showed that muscarinic cholinergic blocker (atropine) or beta-adrenergic blocker (ß-blocker) lowered LF power, enhanced by dual blockade (atropine + ß-blocker) (Akselrod et al., 1981; Pomeranz et al., 1985). Both parasympathetic and sympathetic cardiac activity would therefore be associated with HR power in the LF band. Saul et al. (1990) and others (Pagani et al., 1997) showed a concomitant increase in LF power and muscle sympathetic nerve activity measured by microneurography. Furthermore, under atropine, LF power increased during orthostatic testing (Taylor et al., 1998), and atropine is known to increase sympathetic modulation. Although these studies showed sympathetic cardiac modulation in LF power, changes in LF power can be interpreted only in relation to HF power. Accordingly, normalized indexes such as LF/HF ratio, LF% [LF/(LF + HF)^*^100], and HF% [HF/(LF + HF)^*^100] are used to examine this relationship.

To summarize, whereas HF power is modulated by parasympathetic modulation, LF power is controlled by both sympathetic and parasympathetic activity and normalized indexes allow approaching sympathetic modulation (Pagani et al., 1986; Lombardi and Stein, 2011).

### Non-linear approach: complexity of HRV

Alternatively, non-linear approach was proposed to study cardiac autonomic control (Voss et al., 1995). In the last years, emergent interest of non-linear dynamics that characterize autonomic cardiovascular control lead to a growing literature (Voss et al., 1995; Porta et al., 2007, 2012). The study of the complexity of the different feedback loops impacting on the cardiac function has led to novel indexes capable of reflecting the complexity of the signal. Although several non-linear methods have been developed, we will briefly present entropy-derived measures, which have been recently applied for the assessment of autonomic cardiovascular complexity during sleep such as approximate entropy, sample entropy, corrected conditional entropy and Shannon entropy (Vigo et al., 2010; Viola et al., 2011). The increase the complexity of the cardiac signal, reflected by the increase in these non-linear indexes is usually associated to vagal modulation and its decrease is usually interpreted be the result of an increased sympathetic drive and vagal withdrawal (Porta et al., 2007).

### Time-frequency transforms: transit changes in HRV

Wavelet or Wigner-Ville transforms (Rajendra Acharya et al., 2006) are time-frequency methods used to analyse HR by tracking signal frequency over time. By examining transit changes in LF and HF power and the LF/HF ratio, they describe sympathetic and parasympathetic activity over time, effectively characterizing transit autonomic cardiac changes to short-time tasks (Pichot et al., 1999; Chouchou et al., 2011). These methods allow the study of transient changes in the autonomic nervous system for short periods, from which seconds to minutes. Similarly to evoked-potentials in response to different types of stimuli such as somatosensory, visual or auditory, averaging of several stimuli allows to retrieve a characteristic physiological response of stimuli used. These methods were used during wakefulness to assess autonomic reactivity to tilt tests (Oliveira et al., 2008; Orini et al., 2012) or exercise (Tiinanen et al., 2009) and during sleep to assess autonomic reactivity to periodic leg movements (Sforza et al., 2005), sleep apneas (Chouchou et al., 2014), and experimental pain (Chouchou et al., 2011).

### HRV in human imaging studies

Using only 2–3 skin electrodes (on chest, hands, or feet), HRV provides a simple, easily implemented way to assess ANS activity during in-scanner behavioral, emotional, and sensorimotor tasks known to modulate ANS activity. Fourier transforms, non-linear analysis and temporal analysis are particularly useful for steady-state examination, obtaining a simple index of overall autonomic modulation during imaging (fMRI and PET scan) (Critchley, 2009; Goswami et al., 2011). Time-frequency analysis provides an index of sympathetic and parasympathetic modulation evoked by exteroceptive or interoceptive stimuli for short periods (Sforza et al., 2005; Oliveira et al., 2008; Tiinanen et al., 2009; Chouchou et al., 2011, 2014; Orini et al., 2012). These are simple, non-invasive methods to examine central nervous system activity and identify neural networks involved in autonomic modulation.

## Central and peripheral control of autonomic cardiac activity

**Figure 1:**
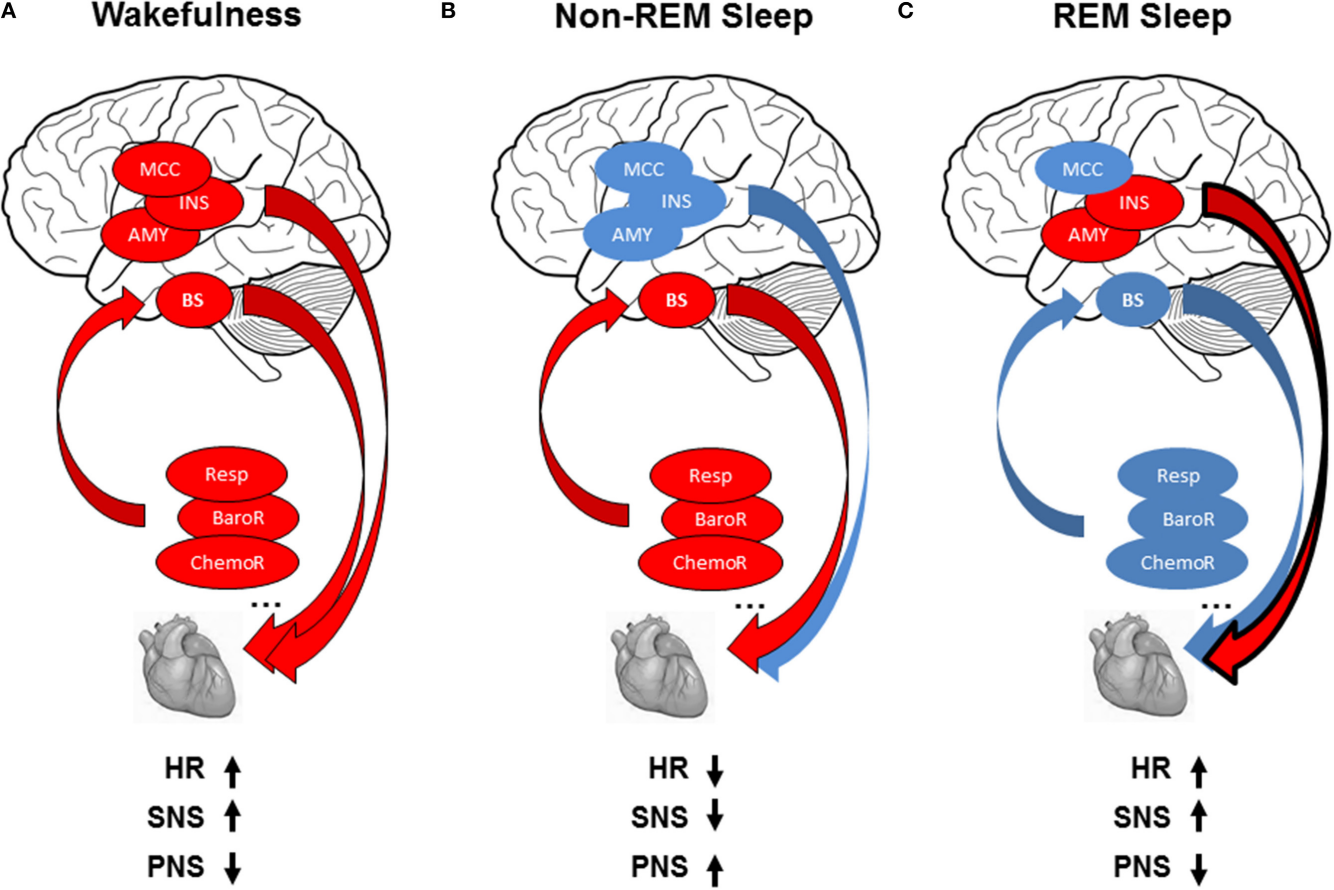
**(A)** Modulation of cardiac activity during wakefulness: reflex loops [baroreflex (BaroR), respiration (Resp), chemoreflex (ChemoR)] including brainstem centers (BS) and central autonomic network including midcingulate cortex (MCC), insula (INS), amygdala (AMY) contribute to cardiac activity, leading to increased heart rate (HR), increased sympathetic activity (SNS), and decreased parasympathetic activity (PNS). **(B)** Modulation of cardiac activity during non-REMS: The drop in brain activity, with predominant contribution of reflex loops on ANS activity, leads to decreased HR, with parasympathetic predominance, and decrease in sympathetic modulation. **(C)** Modulation of cardiac activity during REMS: autonomic cardiac regulation is shared between central control in relation with the insula and amygdala and homeostatic control of the cardiovascular system by reflex loops, leading to decreased HR with sympathetic predominance and decreased parasympathetic activity. Red circles indicate increase and blue circles decrease in autonomic cardiac activity.

Autonomic cardiac activity depends on reflex loops, hypothalamic-brainstem structures, and various somatic and visceral information. Sympathetic activity is underpinned by a neuronal network in the rostral ventrolateral medulla, spinal cord, and hypothalamus (paraventricular nucleus and lateral hypothalamus) (Guyenet, 2013). Parasympathetic activity is underpinned by neurons in the nucleus ambigus and dorsal motor nucleus of the vagus nerve (Ter Horst and Postema, 1997; Pickering and Paton, 2006). These centers receive inputs directly or via the solitary tract nucleus, from stretch-sensitive afferents of ventilation (lung afferents), arterial pressure (carotid and aortic receptors) afferents, muscle receptor afferents (Guyenet, 2013) activated by stretch and metabolites, chemoreceptor afferents activated by hypoxia and hypercapnia, and inputs from somatic and visceral afferents (Guyenet, 2013). Imaging studies showing core brain regions involved in ANS control revealed differential contribution of subcortical and cortical regions to autonomic cardiac control according to autonomic arousal tasks (somatosensory, motor, emotional, cognitive). A “central autonomic network” (CAN) has emerged, reproducible mainly in the amygdala, insular, and midcingulate cortices (Beissner et al., 2013; Critchley and Harrison, 2013) (Figure 1A). Critchley et al. (2003), using cognitive and sensorimotor tasks, showed that LF power positively correlated with changes in neural activity within the anterior cingulate, bilateral insular, hypothalamus, parietal, and somatosensory cortices (LF power was orthogonalised with respect to the HF regressor to remove shared variance within sympathetic and parasympathetic activities in LF power). For parasympathetic modulation, HF power positively correlated with changes in neural activity within the anterior cingulate and bilateral insula cortex and somatosensory cortices. Among others, HF and LF power correlated with neural activity changes within the amygdala, insula, hippocampus, and ventromedial prefrontal cortex for emotional tasks (Lane et al., 2009; Thayer et al., 2012). These observations suggest that the ANS is controlled by different regions involved in identifying, storing, and regulating emotions (Critchley and Harrison, 2013).

Although cardiac activity is largely modulated through reflex loops and hypothalamic-brainstem centers, the CAN appears responsible for rapid changes in behavior-related autonomic activity, particularly sensory, emotional, and cognitive dimensions. The highest levels of sensory and emotional information are integrated by autonomic cardiac activity. Accordingly, HRV could allow an integrated examination of the interactions between peripheral processes of cardiac autonomic modulation reflex loops and central information processing systems, including emotions.

## Parasympathetic cardiac predominance during non-REMS: homeostatic cardiovascular control

**Figure 2:**
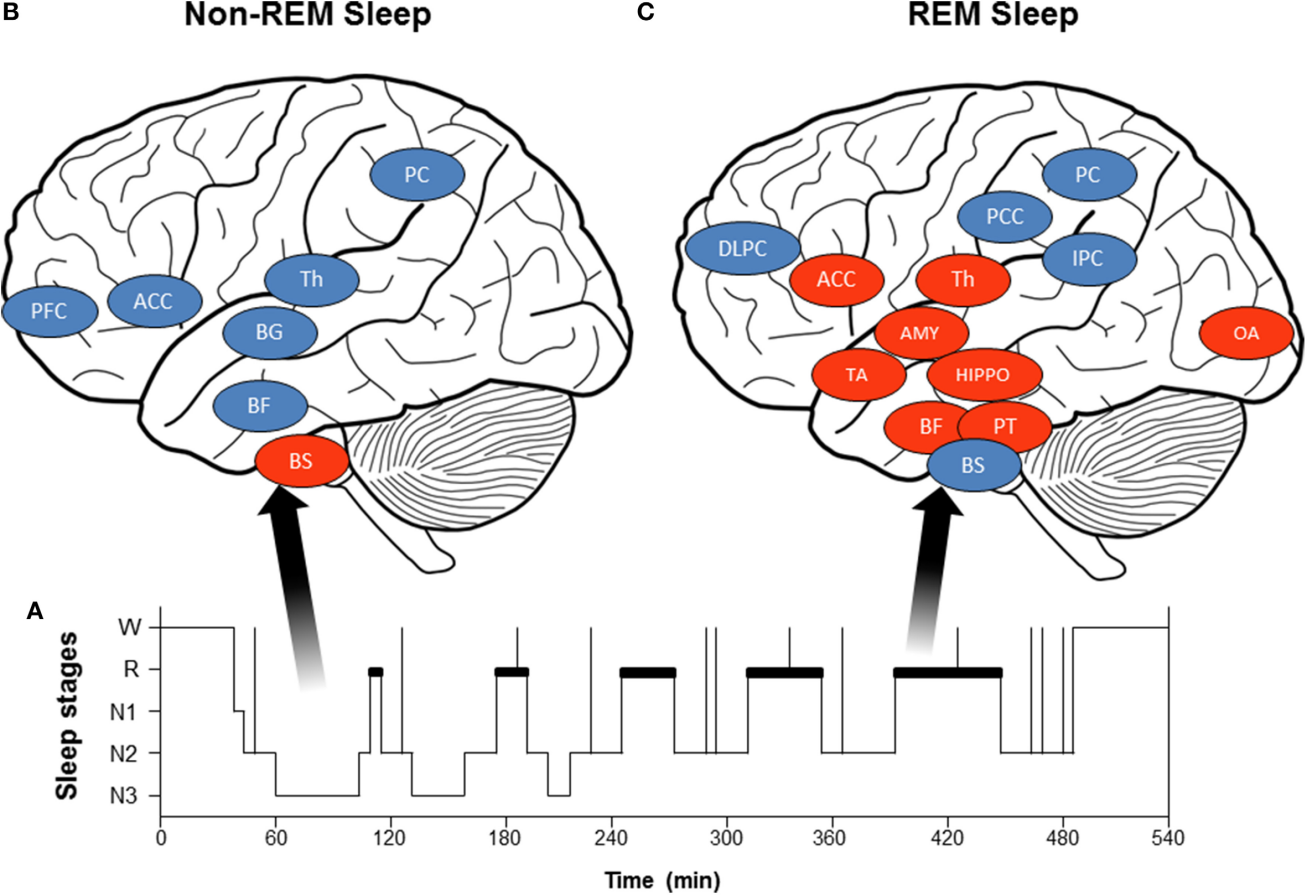
**(A)** Hypnogram (schematic representation) of normal sleep organization: Starting at wakefulness (W), the sleeper begins the night in the lightest sleep stages 1 (N1) and 2 (N2) and progresses to the deepest stages 3 [slow wave sleep (SWS), N3] and REMS (paradoxical sleep or stage R, depicted in bold in the hypnogram). **(B)** Brain activity decreases during non-REMS (blue circles: Th, thalamus; BG, basal ganglia; BF, basal forebrain; PFC, prefrontal cortex; ACC, anterior cingulate cortex; and PC, precuneus) except in brainstem centers (BS). **(C)** Brain activity during REMS: some brain structures show increased activity during REMS (red circles: PT, pontine tegmentum; Th, thalamus; BF, basal forebrain; AMY, amygdala; HIPPO, hippocampus; ACC, anterior cingulate cortex; TA, temporal area; and OA, occipital area), while others become less active (blue circles: DLPFC, dorsolateral prefrontal cortex; PCC, posterior cingulate cortex; PC, precuneus; and IPC, inferior parietal cortex; BS, brainstem).

Humans have three vigilance states: wakefulness, REMS (paradoxical or stage R, according to the American Association of Sleep Medicine, Iber et al., 2007), and non-REMS. Non-REMS is further divided into three stages: from the lightest stages 1 (N1) and 2 (N2) to the deepest stages 3 [slow wave sleep (SWS), N3], defined by electroencephalographic (EEG), electromyographic (EMG), and electroculographic (EOG) characteristics (Iber et al., 2007). The REMS and non-REMS stages string together to form ultradian cycles, which repeat throughout the sleep period (Figure 2A). SWS dominates in the first part, and REMS in the last part.

The wakefulness–sleep transition is accompanied by about a 15% decrease in blood pressure, HR, and cardiac output in normotensive subjects (Smith et al., 1998). HR is markedly decreased when falling asleep and when entering stable non-REMS without arousal (Carrington et al., 2005). These cardiovascular changes are accompanied by increased HF power and decreased LF power and LF/HF ratio, indicating lower cardiac sympathetic modulation with predominant parasympathetic heart modulation (Critchley et al., 2003; Mendez et al., 2006; Lane et al., 2009; Cabiddu et al., 2012; Thayer et al., 2012), and more pronounced in SWS (Van de Borne et al., 1994; Bonnet and Arand, 1997; Carrington et al., 2005) (Figure 1B). The increased complexity of HRV detected during non-REMS study using non-linear indexes also illustrated predominance of parasympathetic control of the heart and sympathetic withdrawal during non-REMS (Vigo et al., 2010; Viola et al., 2011). These HRV-derived changes in cardiac sympathetic modulation are corroborated by studies using other cardiac sympathetic indices such as the cardiac pre-ejection period (Burgess et al., 2004), QT interval (Molnar et al., 1996), muscle sympathetic nerve activity (Somers et al., 1993), and circulating catecholamine concentration (Irwin et al., 1999).

Autonomic cardiac changes during non-REMS may be in relation with global activity and reflex loop changes. First, non-REMS is characterized by slow EEG rhythms accompanied by decreased brain activity compared to wakefulness (Desseilles et al., 2008, 2011b; Dang-Vu et al., 2010; Dang-Vu, 2012), especially in subcortical (brainstem, thalamus, basal ganglia, basal forebrain) and cortical (prefrontal cortex, anterior cingulate cortex, precuneus) regions (Figure 2B). A PET imaging study (Desseilles et al., 2006) found no relationship between brain activity and autonomic cardiac modulation during non-REMS. These studies suggest that decreased activity in subcortical and cortical regions involves a lower central command in cardiac autonomic control. Second, changes in reflex loop activity may contribute to autonomic cardiac modulation changes. Both baroreflex sensitivity (Cortelli et al., 2012) and baroreflex contribution (Silvani et al., 2008) increase during non-REMS (compared to wakefulness), while cardiopulmonary coupling between respiratory frequency and parasympathetic cardiac modulation increases from 8 to 15% (Van de Borne et al., 1995). Altogether, results indicate that parasympathetic predominance and decreased sympathetic modulation during non-REMS is linked to both greater baroreflex and respiratory contributions to ANS activity and decreased brain activity, leading to decreased central modulation of autonomic activity.

In sum, the ANS balance of cardiac control during non-REMS could be due to homeostatic control of the cardiovascular system by ascending visceral information rather than descending cortical information.

## Sympathetic cardiac predominance during REMS

### Central and peripheral cardiovascular system control

Unlike non-REMS, REMS is marked by increased HR, LF power, and LF/HF ratio and reduced HF power, rising toward wakefulness (Van de Borne et al., 1994; Mendez et al., 2006; Cabiddu et al., 2012), showing increased and predominant sympathetic modulation (Figure 1C). Non-linear indexes demonstrated decreased complexity of HRV detected during REMS, also indicating predominance of sympathetic control of the heart and parasympathetic withdrawal during non-REMS (Vigo et al., 2010; Viola et al., 2011). Similar sympathetic cardiac changes were reported in muscle sympathetic nerve activity (Somers et al., 1993) and circulating catecholamine concentration (Irwin et al., 1999). The findings are inconsistent on autonomic cardiac reflexes during REMS: baroreflex was higher compared to non-REMS (Monti et al., 2002; Iellamo et al., 2004), whereas other studies found no difference between REMS and wakefulness (Silvani et al., 2008). Moreover, during non-REMS, respiratory drive regulation was strongly influenced by peripheral inputs, whereas respiration regulation was under central control during REMS (Rostig et al., 2005). However, brain activity patterns during REMS differed from those in non-REMS and wakefulness, with greater activity in certain brain structures during REMS compared to waking (pontine tegmentum, thalamus, basal forebrain, amygdala, hippocampus, anterior cingulate cortex, temporo-occipital areas) and decreased activity in others (dorsolateral prefrontal cortex, posterior cingulate cortex, precuneus, inferior parietal cortex, Figure 2C). Importantly, REMS is also classified into two distinct categories: phasic REM, with rapid eye movements, and tonic REMS, without these movements. Changes in autonomic modulation during REMS are particularly marked during phasic REMS for both heart rate (Coote, 1982) and muscle nerve sympathetic activity (Shimizu et al., 1992). This phasic autonomic activity tends to coincide with eye movements and other events specific to phasic REM, such as theta bursts in the hippocampus (Rowe et al., 1999; Pedemonte et al., 2005). A PET imaging study in humans found a strong relationship between the amygdala, insular cortex, and SDNN of the HRV (Desseilles et al., 2006), indicating strong central control of cardiac modulation during REMS by brain regions known to be involved in ANS modulation during wakefulness.

Thus, autonomic cardiac regulation during REMS appears to be shared between central control (with the insula and amygdala) and homeostatic control of the cardiovascular system through somato-visceral information.

### Autonomic cardiac modulation and dreams

Whereas dreams occur during either REMS or SWS, subjects awakened from REMS reported dreaming 80–85% of the time, vs. only 10–15% when awakened from SWS (Hobson, 1990). REMS dreams are longer, more vivid, bizarre, emotionally intense, and illogical than SWS dreams (Desseilles et al., 2011a). Note that anger and fear are common during dreams, occurring in 57% of all dreams (Merritt et al., 1994).

Importantly, some dream studies investigating the relationship between dream content and autonomic cardiac modulation (Baust and Engel, 1971; Hauri and Van de Castle, 1973) found associations between dream intensity and emotionality and HRV. Hauri and Van de Castle (Baust and Engel, 1971) found strong associations between dream emotionality and intensity during REMS and HRV, and between dream involvement and mean HR. For non-REMS, only mentation intensity and SDNN were related. Accordingly, during REMS, insular and amygdalar interactions involved in cardiovascular regulation (Desseilles et al., 2006) might reflect cortical and subcortical network activity underlying intense emotions, particularly fear and anxiety, often experienced in dreams. More recently, daily worry has been shown to be related to cardiac autonomic changes marked by sympathetic predominance during wakefulness but also during sleep (Brosschot et al., 2007). Overall, these studies suggest that autonomic cardiac modulations during sleep could inform us on sleep mentation and consequently the sleep stages and main brain structures involved.

Moreover, whereas the association between REMS and dreams effectively reduces the characterisation of the neural correlates of dreaming to a comparison between REMS and wakefulness or non-REMS, note that neither dreaming nor REMS are stable, homogeneous, or unique states (Cavallero et al., 1992; Stickgold et al., 2001). Indeed, dreaming can be described along a continuum from thought-like mentation typical of early non-REMS to florid, vivid, and dreamlike experiences typical of REMS. Other studies suggested a shift toward more dreamlike hallucinations and fewer directed thoughts with both REMS duration and total sleep duration (Fosse et al., 2004). These findings suggest that REMS is a facilitating neurophysiological state for dreaming, although dreams are experienced in other sleep stages. This assumption also reflects the importance in emotional memory of noradrenaline (Sara, 2009), which appears to be involved when dream content is especially negative. Finally, progressively increasing sympathetic activity along the sleep duration, independently of sleep stage, was linked to circadian rhythm (Trinder et al., 2001; Carrington et al., 2005). An alternative, complementary interpretation is to link the sympathetic increase to the quality of cognitive dream content during sleep along a continuum: from thought-like mentation in early non-REMS to florid, vivid, dreamlike experiences in REMS, often with fear and anxiety, which might also contribute to sympathetic predominance as sleep progresses.

Along this line, cardiac sympathetic predominance during REMS was studied in several clinical conditions, including REMS behavior disorders (RBD) (Postuma et al., 2010). These disorders are characterized by intermittent loss of REMS atonia, usually seen in REMS, and by elaborate motor activity associated with dream mentation. RBD patients showed autonomic dysfunctions during sleep (reduced tonic and phasic autonomic activity), which tend to appear earlier than autonomic dysfunction during wakefulness (Ferini-Strambi et al., 1996). This result contrasts with the psychophysiological parallels that occur during dreams: sleep mentation during either REMS or non-REMS (Baust and Engel, 1971; Hauri and Van de Castle, 1973). Moreover, given that symptomatic RBD cases were associated with several frequent and debilitating neurological disorders, including the neurodegenerative disorders dementia and Parkinson's disease, and that autonomic dysfunctions might be detected earlier in sleep than in wakefulness and immediately improved by clonazepam (Ferini-Strambi and Zucconi, 2000), HRV measures and analysis during sleep could be used to detect and treat RBD conditions and other potentially co-occurring conditions.

## Implications for psychiatric disorders

HRV analysis could be used to characterize sympathetic and parasympathetic hyperactivity or hypoactivity in many psychiatric disorders (Yeragani et al., 2002; Bär et al., 2007; Kemp et al., 2010). More broadly, anxiety and depressive disorders are associated with sympathetic overactivity, and anxiolytic and antidepressant treatments are known to affect ANS control (Chouchou et al., 2013). Thus, HRV could be a pharmacological or psychotherapeutic aid to reduce the trial and error and side effects of pharmacological treatments designed to restore ANS activity (Spoormaker et al., 2006).

Moreover, emotional states during dreaming may be as intense as during wakefulness (Spoormaker et al., 2006), potentially causing behavioral stress. The ability to perceive emotional state while dreaming is supported by Revonsuo's (Revonsuo, 1995) dreams and virtual realities thesis, and concurs with the observation that lucidly dreamed motor action increases peripheral effector function (i.e., autonomic activity, Erlacher and Schredl, 2008). This suggests that actions and emotions perceived during sleep can modulate cardiac autonomic activity, similarly to during wakefulness. Therapeutically, because wakefulness interventions such as imagery rehearsal therapy (IRT) have been shown to modulate dream mentation during sleep (Krakow and Zadra, 2006), they might also decrease overactive autonomic activity in depression and anxiety, which are frequently associated with negative emotionality during wakefulness and sleep (Beck and Ward, 1961). However, the literature suggests that autonomic changes in depression are linked to antidepressant treatments, and not the disease (Bär et al., 2004). The etiology of cardiac autonomic changes in depression needs to be clarified in order to help prevent associated cardiovascular events (Bär et al., 2004).

Sleep deprivation (SD) involves changes in brain activity in regions that regulate mood and emotions (García-Gómez et al., 2007). These changes can be understood as changes in autonomic modulations, because SD increases stress and anxiety, negative emotions, and sympathetic tone, which cannot by themselves contribute to change brain mechanisms for emotional regulation (Yoo et al., 2007). Interestingly, SD is used to reduce depression in bipolar patients, often combined with light therapy to resynchronise sleep (Bunney and Bunney, 2013). Thus, SD may affect ANS modulation in patients with a deregulated ANS, marked by decreased parasympathetic activity and sympathetic overactivity (Meerlo et al., 2008). The lower energy expenditure following SD and its links to the ANS provide a promising avenue for understanding the positive impact of SD on bipolar depression (Pénicaud et al., 2000; Benedict et al., 2011).

Finally, to better understanding of involvement of cognitions and emotions in autonomic cardiac control, experimental protocols associated with HRV analysis and brain imaging are necessary. These different types of analysis presented in this mini-review provides the possibility for more accurate measurement of interactions between autonomic cardiac activities before and after stimulation and brain processing of somatosensory, visual or auditory stimuli, as well as emotions (Critchley and Harrison, 2013). Furthermore, multivariate signal processing techniques (Porta and Faes, 2013) are particularly relevant for understanding the interactions between the different feedback loops and the influence of higher centers on cardiac function and provide opportunities for better understanding the heart-brain interaction during wake as well as during sleep.

## Conclusion

Brain activity changes during different sleep stages are involved in autonomic regulation, marked by higher parasympathetic tone during non-REMS and sympathetic predominance during REMS. Cardiac autonomic modulation during REMS might partially depend on central nervous system modulation, allowing potential exploration of higher brain structure activity through peripheral autonomic modulation. These are simple, non-invasive methods to study brain activity that could obtain valuable information about emotional states in psychiatric disorders and dream content. However, the precise involvement of higher structures in cardiac autonomic control during REMS remains unclear, along with the link between autonomic modulation and dream content.

### Conflict of interest statement

The authors declare that the research was conducted in the absence of any commercial or financial relationships that could be construed as a potential conflict of interest.
